# Salivary Free Testosterone as a Potential Biomarker for Pelvic Organ Prolapse in Postmenopausal Women: A Prospective Case–Control Study

**DOI:** 10.1111/iju.70473

**Published:** 2026-05-15

**Authors:** Maki Kawasaki, Kei Nagase, Minika Yukimoto, Yukako Yamaguchi, Akihiro Maeda, Shuhei Kusano, Yuka Kakinoki, Hiroaki Kakinoki, Kazuma Udo, Shohei Tobu, Mitsuru Noguchi

**Affiliations:** ^1^ Department of Urology, Faculty of Medicine Saga University Saga Japan

**Keywords:** Dehydroepiandrosterone sulfate (DHEA‐S), enzyme‐linked immunosorbent assay, estradiol, pelvic organ prolapse, salivary free testosterone

## Abstract

**Objective:**

Pelvic organ prolapse (POP) is a common pelvic floor disorder in postmenopausal women, yet no biomarkers currently exist to predict its development. This study aimed to investigate the relationship between sex hormone levels, particularly androgens, and the severity of POP in postmenopausal women.

**Methods:**

We conducted a prospective observational study including 109 postmenopausal women with POP and 66 age‐matched women without POP (control group) at Saga University Hospital. POP severity was classified using the Pelvic Organ Prolapse Quantification (POP‐Q) system. Salivary free testosterone and 17β‐estradiol were measured, along with serum dehydroepiandrosterone sulfate (DHEA‐S), using enzyme‐linked immunosorbent assays. Clinical characteristics and lower urinary tract symptoms were also assessed. Statistical comparisons were performed using t‐tests, Chi‐square tests, and Pearson correlation analysis.

**Results:**

The POP group had significantly higher BMI and parity and reported more severe lower urinary tract symptoms than the control group. Salivary free testosterone levels were significantly decreased, and serum DHEA‐S levels were significantly increased in the POP group (*p* = 0.0157 and *p* = 0.0082, respectively), while estradiol levels showed no significant difference. Advanced POP (stages III–IV) was associated with further reductions in free testosterone. DHEA‐S levels were unexpectedly higher in POP stages II and III compared to controls.

**Conclusion:**

Reduced levels of circulating androgens, particularly Salivary free testosterone, may be associated with POP development and severity. Salivary free testosterone could serve as a non‐invasive biomarker for POP risk stratification. Further longitudinal and multi‐institutional studies are needed to clarify the role of androgens in POP pathophysiology.

## 1 Introduction

Pelvic organ prolapse (POP) is a pelvic floor disorder resulting from the failure of pelvic support structures [1, 2]. Currently, no reliable biomarkers exist to predict the development of POP, and no effective preventive pharmacological therapies are available. Once POP occurs, treatment is limited to physical interventions such as surgery or the use of vaginal pessaries to reposition the prolapsed pelvic organs. In developed countries, the incidence of POP increases with age, especially among postmenopausal women [3]. Although Asian women are reportedly less likely to experience worsening POP symptoms compared to African American, White, and Latina women [4], the estimated number of potential POP patients in Japan exceeds five million. This highlights the urgent need to establish effective preventive strategies for POP in the Japanese population.

Numerous risk factors for POP have been identified, including aging, menopause, parity, high fertility, obesity, levator ani muscle injury, and lifestyle habits that increase intra‐abdominal pressure [5, 6]. Among these, menopause is considered a major contributing factor, and sex hormones are thought to play a critical role in the pathogenesis of POP. Previous studies have primarily focused on estradiol, a key female hormone [7]. Following menopause, estradiol levels decline rapidly, while androgen levels also gradually decrease. Postmenopausal women have significantly lower circulating androgens compared to women in their 20s and 30s, which may contribute to structural and functional changes in the lower urinary tract [8].

Testosterone plays a vital role in maintaining pelvic floor muscle strength and lower urinary tract function through its effects on muscle mass, adiposity, protein synthesis, and urogenital regulation [9].

However, only a few studies have examined the association between circulating androgen levels and POP severity in clinical settings. The existing literature is largely limited to White and African American populations [10, 11], with very few studies involving Asian women.

Given this background, we hypothesized that reduced androgen levels may represent a risk factor for POP in postmenopausal women. The objective of this study was to investigate the relationship between POP severity and circulating sex hormone levels, particularly androgens, and to explore the potential of androgens as predictive biomarkers for POP.

## 2 Materials and Methods

### 2.1 Patient Selection

This prospective study included postmenopausal female patients diagnosed with pelvic organ prolapse (POP group) and postmenopausal patients with urological diseases but without POP (non‐POP group), who visited the Department of Urology at Saga University Hospital in Japan. “Postmenopausal” is defined as 12 months of amenorrhea, excluding women on hormone replacement therapy (systemic or local) or taking hormone‐affecting supplements (such as DHEA or phytoestrogens) excluded.

The control group consisted of women diagnosed with urological conditions such as bladder cancer, urinary stones, and renal tumors. To minimize potential confounding effects of disease activity or therapeutic interventions on sex hormone levels, only patients whose treatment had stabilized were included. The Pelvic Organ Prolapse Quantification (POP‐Q) score, history and factors involved in metabolic syndrome, and blood T‐Chol, LDL‐Chol, and triglyceride levels were extracted from the medical records. Lower urinary tract symptoms were evaluated using the International Prostate Symptom Score (IPSS), IPSS–quality of life (QOL) score, and Overactive Bladder Symptom Score (OABSS). The use of personal information complied with the Personal Information Protection Law and was handled with due consideration for personal privacy and information leakage. All personally identifiable information was excluded. The study was reviewed by the Ethics Committee of the Saga University School of Medicine (Approval No. 2020–09‐R‐07).

### 2.2 Measurement of Sex Hormones

Sex hormone levels were measured using commercially available enzyme‐linked immunosorbent assay kits [9]. Salivary free testosterone and 17β‐estradiol were measured using kits from Salimetrics (Carlsbad, CA, USA; #1–2402 for testosterone, #1–3702 for estradiol), while serum dehydroepiandrosterone sulfate (DHEA‐S) was measured using a kit from Arbor Assays (Ann Arbor, MI, USA; #K054‐H1). Saliva samples were collected in the morning using the Saliva Collection Aid (Salimetrics). Hormone concentrations were quantified using an EnVision plate reader (PerkinElmer Japan, Yokohama, Japan). A preliminary validation confirmed a proportional relationship between serum testosterone and salivary free testosterone levels.

### 2.3 Measure Length of 2nd and 4th Digits (2D/4D Ratio)

Higher testosterone levels have been suggested to be associated with greater testosterone exposure during the embryonic period. The digit ratio of the lengths of the second and fourth digits (2D/4D ratio) is a measure of testosterone exposure during the embryonic period [12]. Higher testosterone exposure during embryonic life results in lower 2D/4D ratios, which are generally lower in males than in females. The 2D/4D ratios were measured to determine the effects of testosterone exposure during the embryonic period (Supplemental Figure S1a).

### 2.4 Statistical Analysis

The data obtained from independent experiments were analyzed using an unpaired two‐tailed Student's *t*‐test (comparisons of two data sets) and Chi‐square or Tukey's HSD test (comparisons of multiple data sets) depending on the results for equality of variance. In this study, associations between variables were evaluated using Pearson correlation coefficients. No multivariable regression analysis was performed. The analyses were performed using JMP Pro 17 for Mac (SAS, Cary, NC, USA). Each value is presented as the mean ± standard deviation (SD). *p*‐value < 0.05 was considered to be a significant finding.

## 3 Results

A total of 175 postmenopausal women were enrolled in this study, comprising 109 patients in the POP group and 66 in the control group. Demographic and clinical characteristics, including age, height, weight, body mass index (BMI), parity, presence of metabolic disorders, and lower urinary tract symptoms, were compared between the two groups (Table 1).

**TABLE 1:** Patient background.

Parameter		POP	non‐POP	*p*
		(*n* = 109)	(*n* = 66)	
Age (years)				
Average (±SD)		70.7 (±7.9)	70.6 (±9.4)	0.9052
BMI (kg/m2)				
Average (±SD)		24.3 (±3.3)	21.9 (±4.0)	< 0.001*
Partiy				
Average (±SD)		02.3 (±0.9)	01.8 (±1.1)	0.0015*
Percentage of metabolic diseases				
Dyslipidemia		30.3% (33/109)	39.4% (26/66)	0.2162
Type 2 diabetes mellitus		16.5% (18/109)	09.1% (6/66)	0.1665
Hypertension		55.1% (60/109)	42.4% (28/66)	0.1056
Non metabolic disease		33.0% (36/109)	43.9% (29/66)	0.1264
One positive above		39.5% (43/109)	27.3% (18/66)	0.1013
Two positives above		20.2% (22/109)	22.7% (15/66)	0.6896
Three positives above		07.3% (08/109)	06.1% (4/66)	0.7337
Biomarkers of metabolic diseases				
T‐Chol	Average (±SD)	192 mg/dL (±34)	195 mg/dL (±44)	0.7188
LDL‐Chol	Average (±SD)	122 mg/dL (±33)	109 mg/dL (±29)	0.0173*
Triglycerides	Average (±SD)	133 mg/dL (±75)	116 mg/dL (±54)	0.8804
Lower urinary symptoms				
IPSS	Average (±SD)	11.6 (±8.4)	6.2 (±6.9)	< 0.001*
IPSS QOL	Average (±SD)	04.2 (±1.8)	2.9 (±1.8)	< 0.001*
OABSS	Average (±SD)	05.6 (±4.0)	3.7 (±2.8)	0.0012*

*Note:* The clinical characteristics of all study participants are shown. Mean values and standard deviations (SD) are shown for continuous variables. The POP group exhibited significantly higher body mass index (BMI), parity, LDL‐Chol levels and scores of lower urinary tract symptom questionnaires (IPSS, IPSS‐QOL, and OABSS) compared to the non‐POP group. The proportion of patients with individual or multiple metabolic diseases did not significantly differ between groups. *p < 0.05 were considered statistically significant.

The POP group showed significantly higher BMI (24.3 ± 3.3 vs. 21.9 ± 4.0, *p* < 0.001) and parity (2.3 ± 0.9 vs. 1.8 ± 1.1, *p* = 0.0015) than the control group. Lower urinary tract symptoms were also more severe in the POP group, as evidenced by significantly higher scores in the IPSS (11.6 ± 8.4 vs. 6.2 ± 6.9, *p* < 0.001), IPSS‐QOL (4.2 ± 1.8 vs. 2.9 ± 1.8, *p* < 0.001), and OABSS (5.6 ± 4.0 vs. 3.7 ± 2.8, *p* = 0.0012). However, no significant differences were observed in the prevalence of diabetes mellitus, hypertension, or dyslipidemia between the two groups.

The distribution of POP‐Q stages in the POP group was as follows: stage I (1.8%), stage II (43.1%), stage III (29.4%), and stage IV (25.7%) (Table 2), indicating that most cases were moderate to severe. In the control group, the most common underlying urological conditions were bladder tumors (51.5%), followed by urinary calculi (19.7%) and renal tumors (16.7%) (Table 3).

**TABLE 2:** Distribution of POP‐Q stages among the POP group.

Variables	
I	01.8% (02/109)
II	43.1% (47/109)
III	29.4% (32/109)
IV	25.7% (28/109)

*Note:* The table shows the proportion of patients with pelvic organ prolapse (POP) classified by the Pelvic Organ Prolapse Quantification (POP‐Q) system. Among the 109 patients in the POP group, the majority were in stage II (43.1%), followed by stage III (29.4%) and stage IV (25.7%), with only 1.8% in stage I.

**TABLE 3:** Underlying urological conditions in the non‐POP group.

Variables	
Bladder tumor	51.5% (34/66)
Urinary stones	19.7% (13/66)
Renal tumor	16.7% (11/66)
Others	12.1% (8/66)

*Note:* This table presents the distribution of primary urological diagnoses among the 66 patients in the non‐POP group. The most common condition was bladder tumor (51.5%), followed by urinary stones (19.7%), renal tumor (16.7%), and other diseases (12.1%).

### 3.1 Sex Hormone Levels in Patients With and Without POP

**FIGURE 1:**
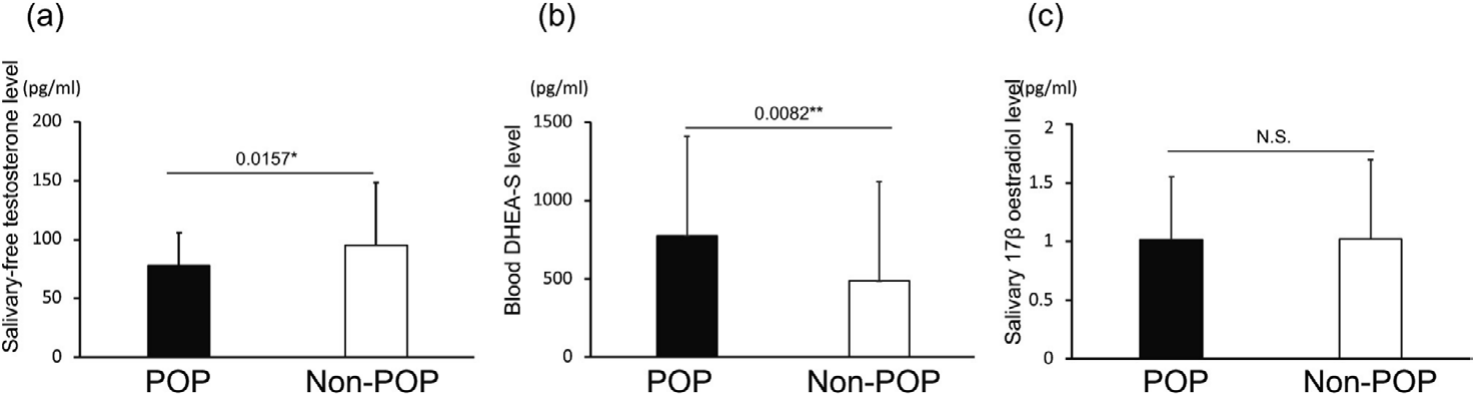
Comparison of hormone levels between women with and without pelvic organ prolapse (POP). (a) Salivary free testosterone levels were significantly decreased in the POP group compared to the non‐POP group (*p* = 0.0157). (b) Blood dehydroepiandrosterone sulfate (DHEA‐S) levels were also significantly increased in the POP group compared to the non‐POP group (*p* = 0.0082). (c) No significant difference was observed in salivary 17β‐estradiol levels between the two groups. Bars represent the mean ± standard deviation. *p < 0.05.

Salivary free testosterone levels were significantly lower in the POP group compared to the control group (78.0 ± 27.8 pg/mL vs. 95.3 ± 53.0 pg/mL, *p* = 0.0157) (Figure 1a). Similarly, serum DHEA‐S levels were significantly increased in the POP group (776.4 ± 632.8 μg/mL vs. 487.0 ± 336.5 μg/mL, *p* = 0.0082) (Figure 1b). In contrast, no significant difference was observed in salivary 17β‐estradiol levels between the two groups (Figure 1c).

### 3.2 Hormone Levels by POP‐Q Stage

**FIGURE 2:**
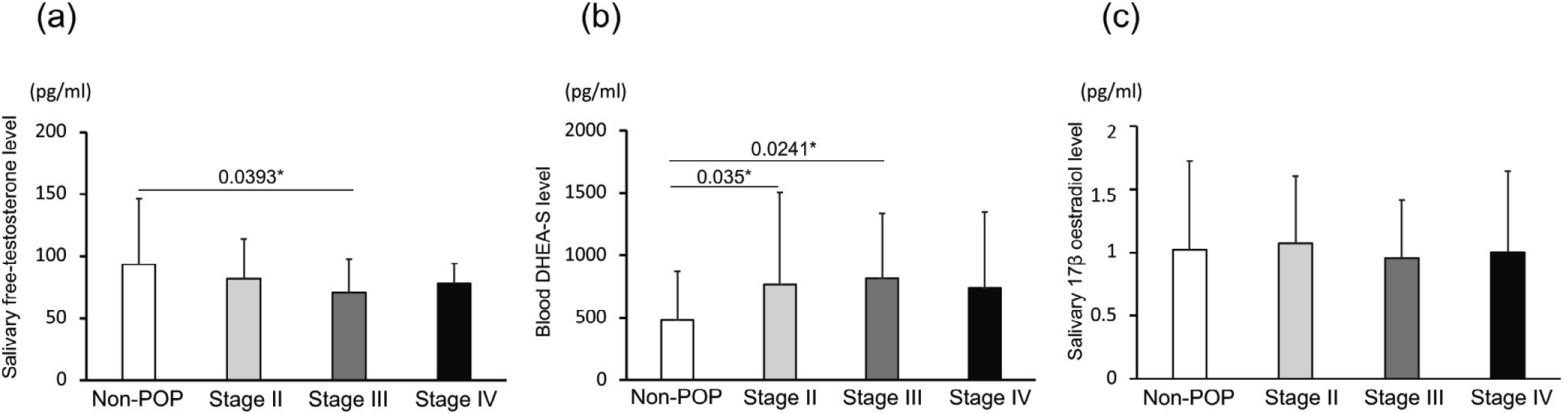
Comparison of hormone levels across different POP‐Q stages and non‐POP group. (a) Salivary free testosterone levels were significantly decreased in women with advanced pelvic organ prolapse (POP‐Q stage III/IV) compared to the non‐POP group (*p* = 0.0393). (b) Blood DHEA‐S levels were significantly increased in the non‐POP group than in POP‐Q stage II (*p* = 0.035) and stage III (*p* = 0.0241). (c) No significant differences were observed in salivary 17β‐estradiol levels across groups. Bars represent mean ± standard deviation. *p < 0.05.

Further analysis according to POP‐Q stage revealed that salivary free testosterone levels were significantly lower in patients with moderate prolapse (stage III) compared to the non‐POP group (*p* = 0.0393) (Figure 2a). Interestingly, serum DHEA‐S levels were significantly higher in patients with stage II (*p* = 0.035) and stage III (*p* = 0.0241) POP compared to controls. In moderate prolapse (II/III), DHEA‐S was a significant increase, but in severe prolapse (IV), it tended to be higher than in the non‐POP group, but no significant difference was observed (Figure 2b). No significant differences in estradiol levels were observed among any POP‐Q stages (Figure 2c).

These findings suggest that circulating androgen levels, particularly salivary free testosterone and DHEA‐S, may be altered in women with POP and that the severity of prolapse is associated with hormonal changes.

### 3.3 Length of 2nd and 4th Digits (2D/4D Ratio)

The 2D/4D ratio was not significantly different between POP (0.985 ± 0.05) and non‐POP (0.973 ± 0.04) patients. No significant differences in the 2D/4D ratios were observed across the POP‐Q stages (stage II: 0.994 ± 0.05; stage III: 0.988 ± 0.04; stage IV: 0.972 ± 0.05) (Supplemental Figure S1b,c).

### 3.4 Correlation Analysis of Factors Associated With POP

**FIGURE 3:**
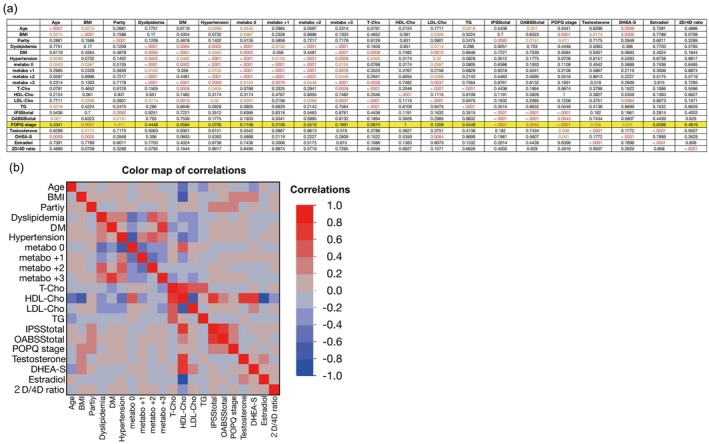
Correlation analysis of factors associated with POP. Metabolic disease was defined as the presence of any of the following: Dyslipidemia, type 2 diabetes mellitus, or hypertension. The variable “metabo” represents the number of these conditions: 0 = none, +1 = one condition, +2 = two conditions, +3 = all three conditions. DM: Type 2 diabetes mellitus; Testosterone: Salivary free testosterone; DHEA‐S: Blood DHEA‐S; Estradiol: Salivary 17β‐estradiol. Factors strongly correlated with a worsening POP‐Q stage were BMI, parity, salivary free testosterone levels, and DHEA‐S levels in blood. (a) *p* value of the correlation. *p* < 0.05 is indicated by red text, and *p* < 0.0001 is indicated by yellow text. The POP‐Q score is indicated with a yellow marker. (b) Color map of the correlations. Darker red indicates a stronger positive correlation, whereas darker blue indicates a stronger negative correlation.

A correlation analysis was conducted on factors related to POP. POP severity demonstrated significant positive correlations with BMI (*r* = 0.31, *p* = 0.0001), parity (*r* = 0.2084, *p* = 0.011), and serum DHEA‐S levels (*r* = 0.1988, *p* = 0.041), while free testosterone (r = −0.1871, *p* = 0.036) was significantly negatively correlated. No significant correlation was found between factors related to metabolism and POP severity (r = −0.0441, *p* = 0.6398). Lower urinary tract symptoms, assessed by IPSS (*r* = 0.3392, *p* < 0.0001) and OABSS (*r* = 0.2356, *p* = 0.0043), showed a significant positive association with POP severity (Figure 3).

## 4 Discussion

In this study, a population of Japan women with postmenopausal pelvic organ prolapse (POP) showed significantly lower salivary free testosterone and significantly higher levels of serum dehydroepiandrosterone sulfate (DHEA‐S) compared to the non‐POP population. Notably, women with advanced POP (POP‐Q stage III) exhibited a more pronounced reduction in salivary free testosterone, suggesting a potential association between POP severity and decreased androgen levels. In the correlation analysis, the factors correlated with POP severity were BMI, parity, salivary free testosterone levels in saliva, and blood DHEA‐S levels.

It is well established that risk factors for POP include aging, menopause, parity, obesity, metabolic disorders, and structural defects of pelvic support tissues [6, 13]. Among these, the decline in female sex hormones after menopause has long been considered a key contributing factor [11]. However, in this study, estradiol levels did not significantly differ between groups, while androgen‐related hormones showed a more distinct relationship with POP status. Even if systemic estradiol levels remain unchanged, local alterations in estrogen receptor biology may still influence pelvic support tissues. Recent findings demonstrate that ERα and ERβ expression levels in pelvic tissues correlate with POP‐Q stage, suggesting that receptor‐level changes, rather than circulating estrogen concentration, better reflect disease severity [14]. Thus, patients with similar salivary estrogen levels may exhibit different local estrogen responsiveness due to receptor imbalance, highlighting the importance of receptor‐dependent mechanisms in POP pathophysiology. These considerations indicate that future studies should incorporate direct assessment of local estrogen receptor expression and signaling in pelvic floor tissues.

We detected a between‐group difference in free testosterone (and DHEA‐S) which Bodner‐Adler's study did not, possibly due to differences in population (Asian vs. Western cohorts), sample size, or the use of salivary free testosterone which reflects bioavailable hormone. These findings suggest that androgens may play a more central role in the development and progression of POP than previously appreciated.

Testosterone and DHEA‐S are known to support protein synthesis, reduce fat accumulation, and maintain skeletal muscle mass [15, 16]. These actions may contribute to the preservation of pelvic floor muscle strength and structural integrity. Both hormones naturally decline with age [17]. Furthermore, androgen receptors have been identified in the pelvic floor and urethral sphincter muscles, supporting their involvement in lower urinary tract function and potentially in the pathogenesis of POP [18].

An unexpected finding in this study was the elevated DHEA‐S levels observed in women with POP‐Q stage II and III compared to the control group.

DHEA‐S levels are known to decrease with age and are reportedly elevated in patients with stress, posttraumatic stress disorder, and depression [17, 19]. Since DHEA‐S is a major adrenal precursor that can be converted into androstenedione and then testosterone, its decline may directly contribute to lower bioavailable testosterone levels. In the present study, the moderate prolapse (II/III) exhibited higher serum DHEA‐S levels despite having lower salivary free testosterone. Although DHEA‐S and testosterone are biochemically linked within the androgen biosynthetic pathway, our data indicate a non‐linear, stage‐dependent pattern, in which testosterone is reduced while DHEA‐S is not uniformly decreased and is elevated in specific stages. Accordingly, interpretations should not assume a monotonic decline in DHEA‐S across all participants but rather a context‐dependent divergence between precursors and downstream androgens.

This result may reflect individual variability in stress responses or endogenous hormonal regulation. These observations underscore the need for future longitudinal studies to clarify the temporal relationship between hormonal fluctuations and POP progression. Salivary free testosterone may be a promising risk marker for POP, but its predictive value needs to be confirmed in future longitudinal studies.

Our findings raise important questions regarding potential clinical applications. For example, salivary free testosterone is easy to measure and could potentially serve as a screening tool; however, it remains unclear at what stage or in which subgroup of postmenopausal women such testing would be most appropriate. One possible scenario might be incorporating this measurement during routine gynecologic examinations in postmenopausal women. Furthermore, the concept of androgen supplementation—whether testosterone or DHEA—as a preventive or therapeutic approach for POP is intriguing but remains experimental. Stronger evidence and well‐designed clinical trials are needed before such interventions can be recommended. In addition, testosterone replacement therapy has been reported to improve muscle strength and quality of life [20], however, this study did not directly assess prolapse outcomes, making its findings suggestive rather than definitive. Future research should therefore explore whether androgen‐based interventions can meaningfully reduce POP risk or progression in randomized controlled trials.

The lengths of the second and fourth digits (2D/4D ratio) are on the order of male<female, as reported by Baker et al. [21]. This ratio is considered an indicator of prenatal testosterone exposure. Higher prenatal testosterone exposure leads to a lower 2D/4D ratio [12]. Women whose fourth digit is longer than their second digit are thought to exhibit more masculine traits, reflecting higher levels of fetal testosterone. The 2D/4D ratio has long been recognized as a marker of prenatal urogenital sex steroid secretion. In this study, the 2D/4D ratio was not significantly different between the POP and non‐POP groups. The findings of the present study suggest that fetal testosterone exposure does not play a role in the development of POP. Postnatal hormonal environment (specifically androgen deficiency in adulthood) is considered to be the focus.

Nonetheless, several limitations must be acknowledged. First, this was a single‐center study, which may limit the generalizability of the findings. Second, hormone measurements in both saliva and serum are susceptible to circadian rhythms and individual variability. It should be noted that sex hormones, particularly testosterone, exhibit diurnal variation, with higher concentrations typically observed in the morning. Although saliva samples in this study were collected during morning hours, the potential influence of diurnal rhythm and intra‐individual variability on hormone levels warrants consideration. These factors may introduce variability that could affect the interpretation of salivary free testosterone as a biomarker. Future studies should aim to standardize sampling times or include multiple time points throughout the day to confirm the robustness and reliability of salivary free testosterone measurements.

Third, the selection of the control group introduces a potential source of bias. Although patients with bladder cancer, renal tumors, and urinary calculi were chosen based on the clear absence of POP and presumed minimal influence on sex hormones, it remains possible that these underlying urological conditions may have affected hormone levels. We believe that future studies should consider including a control group of healthy postmenopausal women without significant comorbidities to ensure that the hormonal differences are truly due to POP status. Therefore, further multicenter, longitudinal studies are warranted to validate our findings and explore the role of androgen‐based interventions in POP prevention and management.

Given that our study population consisted of Japanese women, and previous research in this area has primarily focused on White and African American women, our findings help fill an important gap for Asian populations. Further studies in diverse ethnic groups are warranted to determine whether the observed association between androgen levels and POP is consistent across different genetic and cultural backgrounds. Such efforts will enhance the global relevance of research on POP risk factors.

In conclusion, this study highlights a potential role for androgens in the pathophysiology of POP and provides a foundation for future research on hormonal biomarkers and therapeutic strategies targeting pelvic floor health in postmenopausal women.

## Author Contributions

**Maki Kawasaki:** conceptualization, data curation, project administration, investigation, methodology, visualization, writing – original draft preparation, review and editing. **Kei Nagase:** data curation, project administration, investigation. **Minika Yukimoto:** data curation, investigation. **Yukako Yamaguchi:** data curation, investigation. **Akihiro Maeda:** data curation, investigation. **Shuhei Kusano:** data curation, investigation. **Yuka Kakinoki:** data curation, investigation. **Hiroaki Kakinoki:** resources, investigation. **Kazuma Udo:** resources, investigation, methodology. **Shohei Tobu:** validation, supervision, writing – review and editing. **Mitsuru Noguchi:** funding acquisition, supervision, writing – review and editing.

## Funding

The authors have nothing to report.

## Ethics Statement

Approval of the Research Protocol by an Institutional Reviewer Board: The study protocol was approved by the Ethics Committee of Saga University School of Medicine (2020–09‐R‐07). All procedures involving personal data complied with the Japanese Personal Information Protection Law. Identifiable information was excluded from analysis, and privacy was protected throughout the study.

## Consent

All participants provided written informed consent prior to their inclusion in the study.

## Conflicts of Interest

We would like to disclose that Dr. Mitsuru Noguchi, one of the co‐authors of this manuscript, is an Editorial Board member of the International Journal of Urology. In accordance with the journal's policy, Dr. Noguchi was excluded from all editorial decision‐making processes related to the review and acceptance of this article to avoid any potential conflicts of interest.

## Supporting information


**Figure S1:** iju70473‐sup‐0001‐FigureS1.jpg. **The 2D/4D ratio**.
(a) The ratio between the second and fourth fingers on the dorsal side of the right hand was measured to determine the level of testosterone exposure during the fetal period. (b) The 2D/4D ratio was not significantly different between POP patients and non‐POP patients. (C) The 2D/4D ratio was not significantly different according to the POP‐Q stage. The data are presented as the means ± SDs of 3 measurements.

## Data Availability

Data sharing not applicable to this article as no datasets were generated or analysed during the current study.

## References

[1] J. O. L. DeLancey , “The Hidden Epidemic of Pelvic Floor Dysfunction: Achievable Goals for Improved Prevention and Treatment,” American Journal of Obstetrics and Gynecology 192, no. 5 (2005): 1488–1495, 10.1016/j.ajog.2005.02.028. 15902147

[2] J. O. L. DeLancey , D. M. Morgan , D. E. Fenner , et al., “Comparison of Levator Ani Muscle Defects and Function in Women With and Without Pelvic Organ Prolapse,” Obstetrics & Gynecology 109, no. 2 Part 1 (2007): 295–302, 10.1097/01.AOG.0000250901.57095.ba. 17267827

[3] J. M. Wu , A. F. Hundley , R. G. Fulton , and E. R. Myers , “Forecasting the Prevalence of Pelvic Floor Disorders in U.S. Women: 2010 to 2050,” Obstetrics & Gynecology 114, no. 6 (2009): 1278–1283. 19935030 10.1097/AOG.0b013e3181c2ce96

[4] L. W. Emily , W. Emily , R. Guri , et al., “Racial Differences in Pelvic Organ Prolapse,” Obstetrics & Gynecology 114 (2009): 1271–1277, 10.1097/aog.0b013e3181bf9cc8. 19935029 PMC2879888

[5] S. F. M. Schulten , M. J. Claas‐Quax , M. Weemhoff , et al., “Risk Factors for Primary Pelvic Organ Prolapse and Prolapse Recurrence: An Updated Systematic Review and Meta‐Analysis,” American Journal of Obstetrics and Gynecology 227, no. 2 (2022): 192–208, 10.1016/j.ajog.2022.04.046. 35500611

[6] F. M. V. Tineke , F. M. V. Tineke , W. Mirjam , et al., “Risk Factors for Pelvic Organ Prolapse and Its Recurrence: A Systematic Review,” International Urogynecology Journal 26 (2015): 1559–1573, 10.1007/s00192-015-2695-8. 25966804 PMC4611001

[7] I. M. F. I. Sharif , I. M. F. I. Sharif , B. Christine , B. Christine , H. Suzanne , and H. Suzanne , “Oestrogens for Treatment or Prevention of Pelvic Organ Prolapse in Postmenopausal Women,” Cochrane Database of Systematic Reviews (2010), 10.1002/14651858.cd007063.pub2. PMC13590214 20824855

[8] M. Brzozowska and A. Lewiński , “Changes of androgens levels in menopausal women,” Menopause Review/Przegląd Menopauzalny 19, no. 4 (2020): 151–154. 10.5114/pm.2020.101941 PMC7812536 33488324

[9] A. M. Isidori , E. Giannetta , E. A. Greco , et al., “Effects of Testosterone on Body Composition, Bone Metabolism and Serum Lipid Profile in Middle‐Aged Men: A Meta‐Analysis,” Clinical Endocrinology 63, no. 3 (2005): 280–293. 16117815 10.1111/j.1365-2265.2005.02339.x

[10] B. Bodner‐Adler , K. Bodner , O. Kimberger , K. Halpern , H. Koelbl , and W. Umek , “Association of Endogenous Circulating Sex Steroids and Condition‐Specific Quality of Life Domains in Postmenopausal Women With Pelvic Floor Disorders,” Archives of Gynecology and Obstetrics 297 (2018): 725–730. 29335782 10.1007/s00404-018-4650-7 PMC5808066

[11] B. Bodner‐Adler , K. Bodner , C. Schneidinger , et al., “Pelvic Organ Prolapse and Endogenous Circulating Sex Steroids in Postmenopausal Women: A Case Control‐Study,” European Journal of Obstetrics & Gynecology and Reproductive Biology 210 (2017): 177–181. 28056433 10.1016/j.ejogrb.2016.12.027

[12] E. Ribeiro , N. Neave , R. N. Morais , and J. T. Manning , “Direct Versus Indirect Measurement of Digit Ratio (2D: 4D) a Critical Review of the Literature and New Data,” Evolutionary Psychology 14, no. 1 (2016).

[13] C. Kim , M. Jeon , D. Chung , S. Kim , J. Kim , and S. Bai , “Risk Factors for Pelvic Organ Prolapse,” International Journal of Gynecology & Obstetrics 98, no. 3 (2007): 248–251. 17408669 10.1016/j.ijgo.2007.02.019

[14] D. J. Orlicky , E. E. Smith , R. Bok , et al., “Estrogen and Androgen Receptor Status in Uterosacral Ligaments of Women With Pelvic Organ Prolapse Stratified by the Pelvic Organ Prolapse Histology Quantification System,” Reproductive Sciences 30, no. 12 (2023): 3495–3506. 37430099 10.1007/s43032-023-01283-z PMC10692001

[15] S. Bhasin , L. Woodhouse , and T. W. Storer , “Proof of the Effect of Testosterone on Skeletal Muscle,” Journal of Endocrinology 170, no. 1 (2001): 27–38, 10.1677/joe.0.1700027. 11431134

[16] J. P. Hinson and P. W. Raven , “DHEA Deficiency Syndrome: A New Term for Old Age?,” Journal of Endocrinology 163, no. 1 (1999): 1–5, 10.1677/joe.0.1630001. 10495400

[17] R. F. Greaves , S. A. Wudy , E. Badoer , et al., “A Tale of Two Steroids: The Importance of the Androgens DHEA and DHEAS for Early Neurodevelopment,” Journal of Steroid Biochemistry and Molecular Biology 188 (2019): 77–85, 10.1016/j.jsbmb.2018.12.007. 30557606

[18] M. H. Ho , N. N. Bhatia , and S. Bhasin , “Anabolic Effects of Androgens on Muscles of Female Pelvic Floor and Lower Urinary Tract,” Current Opinion in Obstetrics & Gynecology 16, no. 5 (2004): 405–409, 10.1097/00001703-200410000-00009. 15353950

[19] M. van Zuiden , S. Q. Haverkort , Z. Tan , J. Daams , A. Lok , and M. Olff , “DHEA and DHEA‐S Levels in Posttraumatic Stress Disorder: A Meta‐Analytic Review,” Psychoneuroendocrinology 84 (2017): 76–82, 10.1016/j.psyneuen.2017.06.010. 28668711

[20] J. Tapper , G. Huang , K. M. Pencina , et al., “The Effects of Testosterone Administration on Muscle Areas of the Trunk and Pelvic Floor in Hysterectomized Women With Low Testosterone Levels: Proof‐Of‐Concept Study,” Menopause 26, no. 12 (2019): 1405–1414. 31479032 10.1097/GME.0000000000001410 PMC6893124

[21] F. Baker , “Anthropological Notes on the Human Hand,” American Anthropologist 1, no. 1 (1888): 51–76.

