# Supplementary figures and images for: Salivary Free Testosterone as a Potential Biomarker for Pelvic Organ Prolapse in Postmenopausal Women: A Prospective Case–Control Study
Source: Int J Urol. 2026 May 15;33:e70473. doi: 10.1111/iju.70473 (PMC13177273; doi:10.1111/iju.70473)

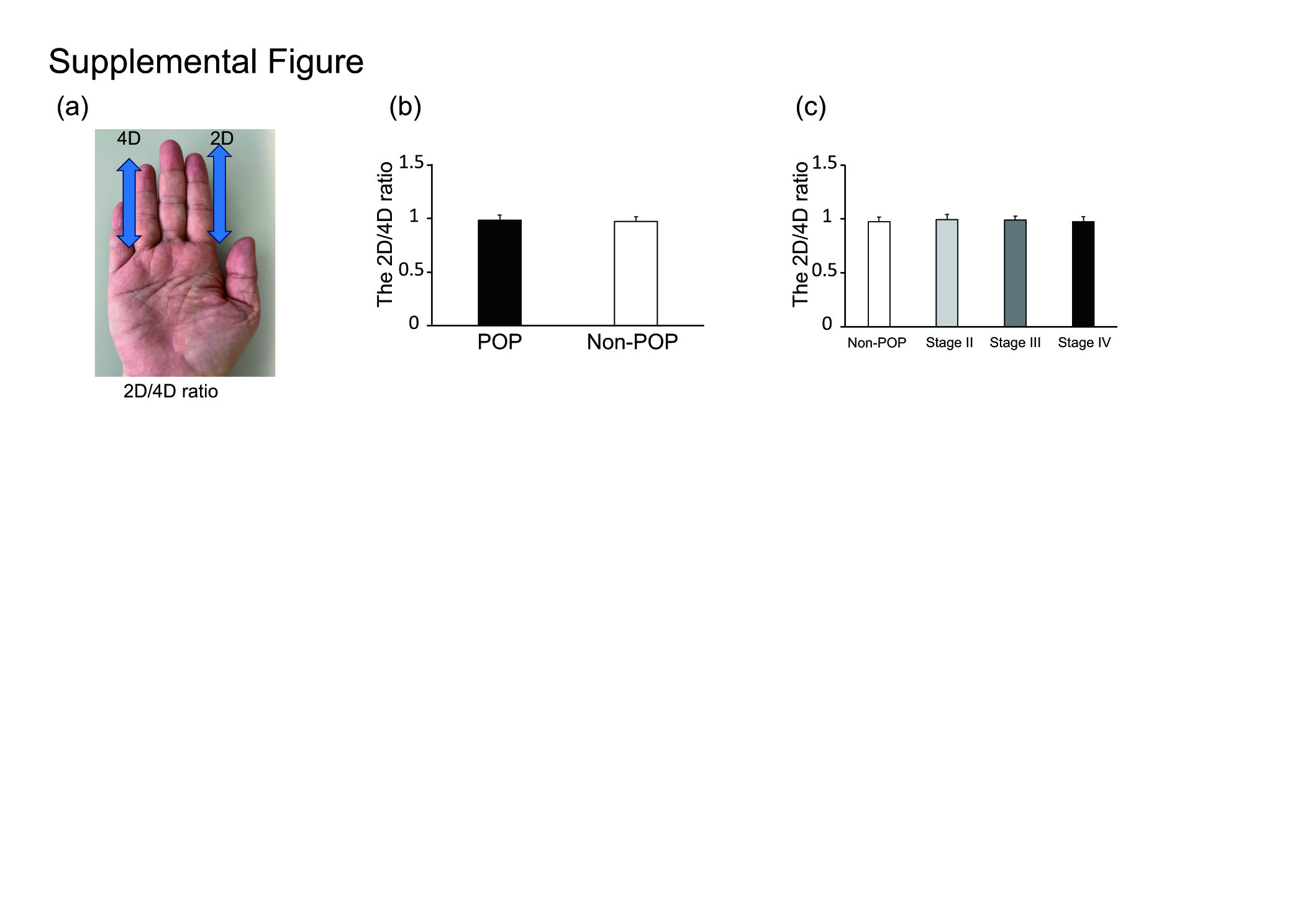

Supplement: Supplementary file 1 — Figure S1: iju70473‐sup‐0001‐FigureS1.jpg. The 2D/4D ratio. (a) The ratio between the second and fourth fingers on the dorsal side of the right hand was measured to determine the level of testosterone exposure during the fetal period. (b) The 2D/4D ratio was not significantly different between POP patients and non‐POP patients. (C) The 2D/4D ratio was not significantly different according to the POP‐Q stage. The data are presented as the means ± SDs of 3 measurements. [file IJU-33-0-s001.jpg]
